# Mr. Koenig, on the Practice of Midwifery in Greece

**Published:** 1801-08

**Authors:** C. Koenig

**Affiliations:** Soho Square


### To Dr. BATTY.

SIR,

THE following account of the ſtate of Midwifery in modern Greece, conſtituting a proof that Nature often defies the moſt deſtructive attempts of art, is tranſlated from a new work of Sonnini, *Voyage en Grèce & Turquie:* If you think this contribution to the hiſtory of the obſtetric art deſerving a place in your uſeful Publication, it is very much at your ſervice.

I am, &c.

*Soho Square, July* 13, 1801. C. KOENIG.

---

THE manner in which the children of the modern Greeks make their firſt entrance into the world is, indeed, too remarkable to be left unnoticed here. It is ſurpriſing, that of the multiplicity of travellers to the Levant, and particularly to the Iſles of the Archipelago, none ſhould have had the leaſt idea of the method theſe people have adopted in aſſiſting at the labours of their women. Having myſelf had an opportunity of being preſent at the delivery of a lady of that country, and being the firſt who has treated upon this ſubject, ſo intereſting for the hiſtory of men, I ſhall not heſitate to enter into the particulars of the manner of their proceedings.

The young woman, at whoſe labour I was preſent, had juſt completed her eighteenth year; ſhe was tall, well made, of a healthy, vigorous conſtitution, and poſſeſſed ſuch a ſhare of beauty, as might have excited the envy of even the antient female inhabitants of Greece. The forerunners of labour manifeſted themſelves juſt when ſhe was going to ſupper; the young lady was, therefore, conducted to her bed-chamber, where I, having obtained permiſſion, did not omit following her. The midwife, who was very old, and reckoned particularly ſkilful in her profeſſion, arrived ſoon after, accompanied by by a female affiftant, who, though equal in age, was far inferior to her in refpect to the expreffion of her features. A painter, wifhing to reprefent a Sybil, could not have felected a better model. Her apparel, and, indeed, the whole of her appearance, was forcerefs like; and the anfwers fhe gave to my queftions, as for their obfcurity, might have paffed for fo many oracles. She alfo brought with her a kind of tripod, compofed of two pieces of wood, joined at an acute angle, and having at the place of their union a flat piece of wood fit for fitting upon; the whole was bound with pieces of old linen, and fupported by three low clumfy legs. The firft attention of the midwife, before fhe began her operation, was directed to the locks; doors, trunks, drawers, and every other thing in the houfe, provided with fuch fafe guards, were carefully opened. This precaution, founded upon a very fingular analogy, is taken with a view to effect an eafy delivery; and from the fame ridiculous principle, matrons only are permitted to attend on thefe occafions, virgins being abfolutely excluded. They likewife informed me, that if I chofe to be prefent, I ought to remain in the chamber till the operation was entirely finifhed, this being a rule from which nobody was permitted to fwerve. The moment the labour begins, thofe who are in the apartment muft not retire, nor are thofe without fuffered to enter; the former are even confidered as impure, and unfit for mixing with other people, until a prieft is called for, who, by beftowing his bleffing, cleanfes them from their fuppofed impurity.

In the mean time Nature began to act; the efforts which fhe ufes for accelerating the birth of a new being became more frequent, and all the circumftances promifed an eafy labour and fuccefsful delivery. During the action of the *infant upon the mother*,* the latter was not fuffered to be at reft, for fhe was forced to walk continually in the apartment; and as often as want of fpirits, or weaknefs, made her wifh for a moment's repofe, the two old matrons would fupport her under her arms, and *oblige* her to continue, though really fhe did not appear to be much pleafed with this *promenade*. When the pains came on, fhe was defired to bend her body forward, while the midwife, being placed behind at the bed, violently preffed the young lady's fides, which was perfevered in till the pains were over. After this a frefh walk commenced, and was continued till new pains afforded an opportunity to the midwife to reaffume her applications.

I am not fufficiently acquainted with the mechanifm Nature employs

* I fcarce need obferve, that this is a miftaken idea of Sonnini.

employs in thefe cafes, to decide whether the method juft related be injurious or not; I can only fay, that it is univerfally practifed in the countries I defcribe, where difficult labour is fcarcely ever witneffed. I may add, that I myfelf beheld the good effects (at leaft apparently) of this operation; for the pains, though rapidly fucceeding one another, were of no long duration, and the young lady feemed little affected by them. A famous phyfician whom I confulted about this, very much difapproved of fuch violent meafures. There is, however, perhaps, no country on the face of the globe where labour is lefs fevere than in Greece: This indulgence of Nature towards the Grecian ladies may be confidered not only as a reward for the fimplicity and regularity of their manner of living, but chiefly as the effect of the climate in which they live: A ferene fky, an atmofphere not condenfed by fevere frofts, but inceffantly warmed by the breath of vernal zephyrs, and impregnated with effluvia's, exciting health and vigour, enable the women to overcome thofe dangers they feem fo perpetually liable to.

Man-midwives are quite unknown in this country, and really if any fhould attempt to practife here, he would meet with no encouragement; for, without having read *Hacquet's* work, they think it the higheft degree of indecency for a woman to employ a man for her delivery: And really Nature in this country performs every requifite herfelf, while the midwives, on the other hand, ufe every means to counteract it. In cafes of fome difficulty, they will refort to fuperftitious remedies; (with them fcience does not extend farther than that) but fortunately, fuch cafes are ranked among things extraordinary.

During the time I fpent in the chamber of the young Grecian in labour, I afked the midwife feveral queftions concerning her practice; for inftance, I enquired what fhe ufed to do in cafes of an unnatural pofition of the child? Such cafes, fhe replied, happened but very feldom; if they did, fhe would endeavour to bring the infant into due pofition; and this proving fruitlefs, fhe would apply to the hufband, who, in the opinion of the women of this country, has it fully in his power to remove every obftacle to a fuccefsful delivery. This magic power, which, fhe affured me, would never fail, confifts in three raps, which the hufband applies with his fhoe upon the fhoulders of his wife, pronouncing at the fame time, with a loud voice, the following words: "'Tis I who have given you this burden; 'tis I who take it off."

At laft the critical moment arriving, the young woman was placed upon the tripod; good nature and apprehenfion were depictured upon her face, and the placidity of her features appeared not to be much altered by the pains ſhe endured. The midwife placed herſelf before her, rather lower, and the aſſiſtant ſitting behind the patient upon a more elevated chair, flung her arms round the middle of her.

The infant ſoon made its appearance: When it was ſeparated from the placenta, the aſſiſtant, with her vigorous arms, lifted the patient for ſeveral times, perpendicularly, over the tripod, upon which ſhe again dropped her with great rudeneſs. In this manner ſhe was unpitifully handled till the delivery was entirely concluded, which fortunately took place very ſoon.

However ſolid the motives may be by which rational phyſicians are prompted to exclaim againſt ſuch a rude method of haſtening delivery, yet it would be a matter of great difficulty to aboliſh it in a country where they do not experience any fatal conſequences attending it. It was aſtoniſhing to me, that the patient herſelf did not at all complain of ſuch a cruel procedure, but went to bed without any appearance of fatigue. A very ſhort repoſe rendered her as eaſy as ſhe was before; her complexion, though (now) leſs brilliant, ſtill retained its former freſhneſs: ſhe received, without conſtraint, a volley of congratulations, and anſwered them as if her ſituation was the moſt tranquil imaginable.

Directly after her delivery, the lady was cloſely wrapped from her boſom to the flanks in a broad linen bandage. Here again the European phyſician will have an opportunity to cavil at the application of bandages: "Every mechanical compreſſion of the abdomen of a delivered woman is highly pernicious; the weight of the infant, during nine months of pregnancy, the ſucceſſive contractions of the uterus, or the violent pains of labour, having co-operated to irritate the organs; every compreſſion in this ſtate cannot but be extremely injurious."* Though this reflection may be the reſult of a rational theory, yet the ladies of Greece would conſider it as merely chimerical; for, indeed, they ſuffer the preſſure of the bandage with the *ſame impunity with which they defy* the dangers of the violent treatment in the firſt ſtage of labour. They even pretend to derive from this operation the advantage of preſerving the beauty of their forms, by *preventing* the uſual concomitants of frequent labours, an exceſſive ſwelling, and the appearance of wrinkles on the abdomen.

During the firſt day after delivery, the midwife boils freſh petals of roſes in wine and honey, and afterwards uſes the ſame decoction

* From a letter written by *Saccombe* to *Sonnini*, on the danger attending the Grecian practice of midwifery.

decoction from the dried leaves. After feveral lotions with the former, the ufe of the latter is continued till the next day, when the pudenda are fomented with cotton dipped in warm wine, after which, the powders of cinnamon or cloves, nutmeg or carraway feed, are applied alternately; one of thefe aromatic fubftances being only ufed at a time, and changed at each dreffing.

Inftead of wine, which is only ufed for the above mentioned purpofe in cafes of great delicacy of the frame, they generally refort to brandy, the application of which is not without a great fhare of pain. Whatever be the ftate of the delivered woman, the dreffing with the aromatic fubftances is continued for the fpace of eight days, morning and evening. The moft fingular circumftance is, that the midwife, at each dreffing, afcends the bed at the fide oppofite to the pillow, and having placed her legs between thofe of the patient, fhe takes hold of her hands, and putting one of her feet exactly upon the fuffering parts, fhe fhocks them for three times with the greateft violence.

On the evening of the eighth day, an egg is boiled hard, which, after being deprived of its fhell, and ftrewed over with the powder of one of the mentioned fpices, is tied to thofe parts which had experienced the rude foot of the midwife, and left there for two or three hours. This operation, which, as the old matron gravely told me, is made with a view to remove the cold which the patient might have poffibly caught, puts a final end to the treatment after the delivery, and the midwife is difmiffed.

